# Selective inhibition of brain endothelial Rho-kinase-2 provides optimal protection of an *in vitro* blood-brain barrier from tissue-type plasminogen activator and plasmin

**DOI:** 10.1371/journal.pone.0177332

**Published:** 2017-05-16

**Authors:** Be’eri Niego, Natasha Lee, Pia Larsson, T. Michael De Silva, Amanda E-Ling Au, Fiona McCutcheon, Robert L. Medcalf

**Affiliations:** 1 Molecular Neurotrauma and Haemostasis, Australian Centre for Blood Diseases, Monash University, Melbourne, Victoria, Australia; 2 Wallenberg Laboratory, Department of Molecular and Clinical Medicine, Institute of Medicine, Sahlgrenska Academy, University of Gothenburg, Gothenburg, Sweden; 3 Department of Physiology, Anatomy and Microbiology, School of Life Sciences, La Trobe University, Melbourne, Victoria, Australia; Massachusetts General Hospital, UNITED STATES

## Abstract

Rho-kinase (ROCK) inhibition, broadly utilised in cardiovascular disease, may protect the blood-brain barrier (BBB) during thrombolysis from rt-PA-induced damage. While the use of nonselective ROCK inhibitors like fasudil together with rt-PA may be hindered by possible hypotensive side-effects and inadequate capacity to block detrimental rt-PA activity in brain endothelial cells (BECs), selective ROCK-2 inhibition may overcome these limitations. Here, we examined ROCK-2 expression in major brain cells and compared the ability of fasudil and KD025, a selective ROCK-2 inhibitor, to attenuate rt-PA-induced BBB impairment in an *in vitro* human model. ROCK-2 was highly expressed relative to ROCK-1 in all human and mouse brain cell types and particularly enriched in rodent brain endothelial cells and astrocytes compared to neurons. KD025 was more potent than fasudil in attenuation of rt-PA- and plasminogen-induced BBB permeation under normoxia, but especially under stroke-like conditions. Importantly, only KD025, but not fasudil, was able to block rt-PA-dependent permeability increases, morphology changes and tight junction degradation in isolated BECs. Selective ROCK-2 inhibition further diminished rt-PA-triggered myosin phosphorylation, shape alterations and matrix metalloprotease activation in astrocytes. These findings highlight ROCK-2 as the key isoform driving BBB impairment and brain endothelial damage by rt-PA and the potential of KD025 to optimally protect the BBB during thrombolysis.

## Introduction

Thrombolysis with recombinant tissue-type plasminogen activator (rt-PA) continues to serve as the primary treatment option for acute ischaemic stroke (AIS) within a confining therapeutic window of 3–4.5 h from stroke onset [1, 2]. Other major limitations, such as poor efficacy for large clots and large vessel occlusions as well as increased risk for development of lethal symptomatic intracerebral haemorrhage (sICH) [2, 3] translate into a low percentage (≤10%) of eligible patients and fuel ongoing search for therapeutic improvement [1, 2]. More than a decade of research has indicated that rt-PA also has off-target effects in neurons, glia [4, 5], but especially at the blood-brain barrier (BBB) [6], where it compromises BBB integrity in a variety of plasmin-dependent and independent mechanisms [7, 8]. Since disruption of the BBB by rt-PA could contribute to brain oedema and sICH, blockade of rt-PA-triggered damaging pathways became the focus of many studies aiming to improve the safety of thrombolysis [7, 8].

Rho-kinase (ROCK), a downstream effector of the small GTPase Rho, is a serine/threonine kinase [9, 10] whose activation has been linked to detrimental sequelae in many cardiovascular conditions [11, 12], including stroke [13]. Pharmacological inhibition of ROCK improves vascular outcomes (e.g. smooth muscle contractility, cerebral blood flow, oedema, endothelial inflammation and neutrophil infiltration) and neurological consequences (infarct size, neurological deficit) in several animal models of focal cerebral ischaemia [10–12, 14–18] as well as in clinical stroke trials [12, 19].

Importantly, the ROCK pathway has also been associated with thrombolysis and BBB breakdown, as we have previously identified *in vitro* that rt-PA affects the BBB via plasmin-dependent activation of ROCK in astrocytes, leading to their retraction [20]. rt-PA further controls ROCK-mediated upregulation of matrix metalloproteinases (MMP)-2 and -9 in astrocytes [21] and brain endothelial cells (BECs) [22] while plasmin cleavage of monocyte chemoattractant protein (MCP)-1 enhances ROCK and ezrin-radixin-moesin (ERM)-dependent tight junction disruption [23], activities which contribute to BBB failure. Fasudil, a nonselective ROCK inhibitor (below), protected the BBB from rt-PA and plasmin *in vitro* [20] and decreased thrombolysis-associated BBB breakdown, MMP activation, haemorrhagic transformation, mortality and neurological deficit *in vivo* [22, 24]. The ROCK pathway therefore represents an excellent candidate for targeted inhibition during thrombolysis in AIS, a strategy which may generally protect the brain from the ischaemic attack and simultaneously preserve the BBB from the actions of rt-PA and plasmin.

Inhibition of ROCK alongside rt-PA may be hindered, however, by specificity caveats. Two isoforms of ROCK have been described, ROCK-1 (p160ROCK; ROKβ) and ROCK-2 (ROKα) [10, 11]. ROCK-1 is ubiquitously expressed (with low levels in brain and muscle), while ROCK-2 is abundant in the brain [25, 26], particularly in neurons [25, 27] and in reactive astrocytes [27, 28]. The two isoforms share 65% overall amino-acid sequence identity and particularly high homology in the kinase domain (92%) [26, 29]. These attributes initially led to their view as functionally redundant [29], yet recent studies using short interfering RNA (siRNA) identified more distinct and sometimes opposing isoform activities in a number of cell-types, including neurons, fibroblasts, smooth muscle cells, immune cells and endothelial cells [11, 30–38]. The most widely used ROCK inhibitors, namely Y27632 (a potent inhibitor of both ROCK isoforms) [39] and fasudil hydrochloride (HA1077, which displays the highest affinity towards ROCK-2) [39] are not entirely isoform-specific [15, 40] or even ROCK-specific and block at similar concentrations a wider range of kinases, for example protein kinase C-related kinase (PRK) 1 and 2 [10, 39]. A more selective blockade may be beneficial to avoid undesired systemic side-effects of ROCK inhibition, for instance hypotension and reduction in resting cerebral blood flow, which may lead to brain hypo-perfusion seen previously in mice with fasudil and Y27632 during stroke [41]. Furthermore, detrimental effects of rt-PA and plasmin in cultured BECs were poorly blocked by fasudil in our previous studies [20], raising the need for closer examination of rt-PA-induced ROCK engagement in the brain endothelium.

More recently, the selective ROCK-2 inhibitor KD025 (formerly known as SLx-2119) was introduced. KD025 is ~200-fold fold more selective for ROCK-2 than ROCK-1 [15, 40]. Encouragingly, KD025 is well-tolerated in healthy men [38] and in a mouse model of stroke it was found to be at least as efficacious as nonselective ROCK inhibitors for reduction of infarct size and neurological deficit without induction of significant hypotension [15]. These features potentially make KD025 a more suitable agent for antagonism of rt-PA-mediated BBB disruption during thrombolysis. Nevertheless, the specific involvement of ROCK-2 in BBB and brain endothelial function as well as in the action of rt-PA has not been studied and KD025 is yet to be examined in these contexts.

Here, we tested in an *in vitro* model of the human BBB whether KD025 can preserve the BBB during rt-PA and plasmin attack under both normal and stroke-like conditions. We report a potent protective effect of selective ROCK-2 inhibition by KD025 on the rt-PA-affected BBB, which is superior to the action of nonselective inhibition by fasudil. Furthermore, we suggest that the ROCK-2 isoform is distinctly involved in rt-PA-induced impairment of brain endothelial cells, making KD025 an exciting candidate for further *in vivo* studies to improve thrombolysis in AIS.

## Materials and methods

### Reagents

Human t-PA (rt-PA; Actilyse^®^) was purchased from Boehringer Ingelheim GmbH (Rhein, Germany) and dialysed against 0.4M HEPES pH 7.4 to remove the original vehicle components [5]. Human Glu-plasminogen was from Enzyme research laboratories (South Bend, IN, USA). Fluorescein isothiocyanate (FITC)-conjugated BSA, bovine aprotinin and endothelial cells growth supplement (ECGS) were obtained from Sigma Aldrich (St Louis, MO, USA). HA1077 (fasudil; hydrochloride) was purchased from Cayman Chemicals (Ann Arbor, MI, USA) while KD025 (SLx-2119) from MedChem Express (Princeton, NJ, USA). Matrigel was obtained from BD Biosciences (Australia) and rat collagen type-I from Trevigen (Gaithersburg, MD, USA). All TaqMan^®^ gene expression assays and reagents were purchased from Applied Biosystems (Thermo Fisher Scientific, Australia).

### Cell culture

#### Human cells

Human transformed SVG astrocytes [42] and primary human brain microvascular endothelial cells (BECs; ACBRI 376, Cell Systems, Kirkland, WA, USA) were cultured as described [20], using Endothelial Cell Growth Media 2 with SupplementMix (Promocell, Heidelberg, Germany) as the BECs culturing medium.

#### Primary mouse cells

All animal procedures were undertaken in accordance with the National Health and Medical Research Council (NHMRC) “Code of Practice for the Care and Use of Animals for Experimental Purposes in Australia” and were approved by the Alfred Medical Research and Education Precinct Animal Ethics Committee (AMREP AEC) of Monash University. Animal procedures also complied with the ARRIVE guidelines (Animal Research: Reporting in Vivo Experiments). Primary mouse brain endothelial cells (mBECs) were prepared from brains of 3–5 weeks-old C57Bl/6 male mice according to our published method [20], without the cell strainer filtration step for removal of larger vessels. mBECs were grown in Endothelial Cell Growth Media 2 with SupplementMix (PromoCell) on Matrigel-coated wells and were typically ready for experimentation on Days *In Vitro* (DIV) 4–6. Primary mouse astroglial cells were prepared from whole brains of 1-day-old C57Bl/6 mice as described [20]. These glial cultures contained primarily astrocytes (identified by immunostaining for GFAP) and ~15% microglia (detected by CD11b immunostaining) [20]. Cultures of primary cortical neurons were prepared from C57Bl/6 mouse embryos at E15-16 according to Samson *et al*. 2008 [5] and used for experimentation on DIV 7–9.

### Real-time RT-PCR

Cells were cultivated in their respective complete medium until confluent or for the specified time (for the neuronal cultures) and then harvested in RLT lysis buffer (Qiagen, Hilden, Germany) for RNA extraction. DNA-free RNA was prepared using RNeasy Mini Kit and DNase I set (Qiagen) according to manufacturer’s protocols. Each culture was tested in duplicate and at least 3 independent cultures were prepared.

Identical amounts of total RNA were reverse-transcribed (1μg or 0.25μg of each human cell or mouse cell sample, respectively) and real-time PCR was performed as previously described [43]. Human ROCK-1 and ROCK-2 were detected using gene expression assays Hs01127699_m1 and Hs00178154_m1, respectively; while mouse ROCK-1 and ROCK-2 were detected using assays Mm00485745_m1 and Mm01270843_m1, respectively. Human and mouse hypoxanthine-guanine phosphoribosyl transferase (HPRT) (gene expression assays Hs99999909_m1 and Mm01545399_m1, respectively) were used as endogenous internal standards. To ensure a valid comparison between ROCK-1 and ROCK-2 we verified the PCR efficiency of each ROCK gene expression assay on an RNA dilution series (S1 Fig). Since the PCR efficiency range was 1.94–2.04, a value of 2 was used in all analyses.

### A human blood-brain barrier model

An *in vitro* model of the BBB was prepared according to our protocol [20, 44] as a co-culture system of SVG astrocytes and human BECs seeded on the opposite surfaces of a collagen-I-coated porous membrane. 4 x 10^4^ SVGs were first permitted to adhere for 4 h onto the underside of an inverted Transwell^®^ insert (polyester membrane, 6.5mm diameter; 3μm pore size; Corning) before seeding 2 x 10^4^ BECs in the inner well. The co-cultures were maintained in BECs growth medium for three days in a humidified 37°C incubator at 5% CO_2_ and 21% O_2_. The contribution of each cell type to permeability is this model has been well-characterised (reference [20], supplemental information).

For experimentation, both the luminal and abluminal chambers were washed once with serum-free medium (phenol red-free DMEM/F-12 with 15mM HEPES (Thermo Fisher Scientific) supplemented with 50μg/ml gentamycin, 1% (v/v) DMSO (to control for the KD025 solvent) and, for 24 h experiments only, also with 20μg/ml heparin and 20μg/ml ECGS). The luminal medium was than replaced again with serum-free medium containing rt-PA and plasminogen at the indicated concentrations. Fasudil (prepared in PBS) and KD025 (prepared as a 100x stock in DMSO) were added simultaneously into both endothelial and astrocytic compartments. To determine changes in BBB permeability after treatment, media from the luminal well was supplemented with 0.35% (w/v) FITC-conjugated BSA. Fluorescence in the abluminal chamber was measured 1 h later using a micro-plate reader (FLUOstar Optima, BMG labtech, Australia). Data is presented as fold change in passage of FITC-BSA relative to DMSO-treated control inserts [20].

### Oxygen-glucose deprivation (OGD)

To stimulate cells under stroke-like conditions, mono-cultures and co-culture inserts were washed once and the medium replaced with serum- and glucose-free DMEM/F12 medium (US Biological, MA, USA) supplemented with 50μg/ml gentamycin and 1% (v/v) DMSO. Stimulating agents were then added as stipulated above for experiments under normoxia. Next, cells were transferred to a hypoxic chamber (Modular Incubator Chamber; Billups-Rotheburg, Del Mar, CA, USA) which was flushed (6 min) with a gas mixture containing 95% nitrogen and 5% CO_2_. The hypoxic chamber was kept humidified at 37°C for the duration of the experiment. FITC-BSA permeability assay (above) was then performed over 1 h under normal atmospheric conditions.

### Phase-contrast microscopy and immunocytochemistry

BECs, SVGs or primary mouse astrocytes were cultured to confluence in 24-well plates (for phase imaging) or in μ-slides 8-wells (Ibidi, Munic, Germany; for immunofluorescence). Wells were coated with gelatine or poly-D-lysine (PDL) for BECs or mouse astrocytes, respectively. Cells were treated in the respective serum-free medium with rt-PA and plasminogen, with or without KD025 as stipulated. They were then gently washed with PBS and fixed for 20 min with ice-cold 4% paraformaldehyde (PFA). Phase-contrast images were captured using a Nikon Eclipse TS100 inverted microscope equipped with objectives Plan Fluor 4x magnification air, 0.13 numeric aperture (NA); Plan Fluor 10x air, 0.3 NA; S Plan Fluor ELWD 20x air, 0.45 NA; and a Nikon DS-Fi2 digital camera with Nikon NIS-Elements F version 4.00.00 software.

Immunocytochemistry was performed according to our published protocol [20] using a rabbit anti-human zonula occludens 1 (ZO-1) antibody applied overnight at 4°C (diluted 1:100) and an Alexa Fluor 568-conjugated goat anti-rabbit IgG (3h at 4°C, diluted 1:1000). Filamentous actin was stained with Alexa Fluor 568 phalloidin (2h at 4°C, diluted 1:500). Hoechst (5 mg/ml) was used as a nuclear counterstain (all antibodies and reagents were from Thermo Fisher Scientific). Fluorescent images were captured on a Nikon Eclipse T*i*-E motorised inverted microscope equipped with an Apo LWD 40x water, 1.15 NA λS objective and an Andor Zyla sCMOS camera with Nikon NIS-Elements software as mentioned above.

### Western blotting

For detection of ROCK-1 and ROCK-2 proteins, whole cell lysates of untreated cells were prepared using ice-cold Radioimmunoprecipitation Assay (RIPA) buffer containing protease and phosphatase inhibitors (Roche applied science, Australia). 22μg and 7μg total protein of human cells and mouse cells, respectively, were subjected to western blot analysis as previously described [5, 20] using a rabbit monoclonal anti-ROCK-1 and a rabbit polyclonal anti-ROCK-2 antibodies (both from Cell Signaling Technology). Mouse anti-β tubulin (D66) (Sigma Aldrich) and rabbit anti-mouse Heat Shock Protein 90β (Merck Millipore) served as loading controls.

For measurement of myosin light-chain phosphorylation, sub-confluent mouse glial cultures in a PDL-coated 12-well plate were washed once and equilibrated at 37°C for 4 h in serum-free medium before addition of DMSO, rt-PA+plasminogen or KD025 directly to the medium for 2 h. Whole cell lysates were then prepared as described above with addition of 10μM aprotinin to the lysis buffer. Phosphorylated and total myosin light-chain 2 (MLC) levels were detected in equal sample volumes (33μl; ~10μg total protein) by rabbit polyclonal anti-pMLC 2 (Ser19) (Cell Signaling Technology) and by mouse monoclonal anti-MRCL3/MRLC2/MYL9 [E-4] (Santa Cruz Biotechnology) antibodies, respectively, and quantitated by densitometry using ImageJ.

### Gelatine zymography

MMP-2 and -9 activity in conditioned medium was detected by electrophoresis of serum-free media samples (20–50μl) loaded under non-denaturing conditions onto SDS-polyacrylamide gels containing 0.03% (w/v) gelatine. Following 3 washes with Triton X-100 solution (2.5% v/v) to remove SDS, gels were incubated overnight at 37°C in developing buffer (10mM CaCl_2_, 50mM Tris-HCl, pH 7.4, 150mM NaCl and 1% (v/v) Triton X-100) with gentle agitation. To inhibit MMP activity, the broad-spectrum MMP inhibitor GM6001 (10μg/ml) was added to the developing buffer. Bands of gelatinolytic activity were visualised by Commassie blue staining and quantitated by densitometry using ImageJ software.

### Cell death and cell viability assays

BECs and SVG astrocytes were grown to confluence in 24-well plates and stimulated in serum-free medium for 6 h or overnight (17 h). Cell death and cell viability were then determined by the lactate dehydrogenase (LDH) release assay (CytoTox 96^®^, Promega, Madison, WI, USA) and by the methyl-thiazole-tetrazolium (MTS) assay (CellTiter 96^®^, Promega), respectively, according to the manufacturer’s instructions. Etoposide (100μg/ml) served as a positive control for cytotoxicity.

### Statistical analysis

Statistical analysis was performed using GraphPad Prism 6 software. Each experiment was typically conducted in duplicate or triplicate and at least three independent experiments were performed. Comparison of experimental data sets was performed by one- or two-way ANOVA with Tukey’s or Newman-Keuls post-hoc correction, while differences between two groups were assessed by two-tailed student t-tests (paired or unpaired). Probability values under 0.05 were considered significant.

## Results

**Fig 1:**
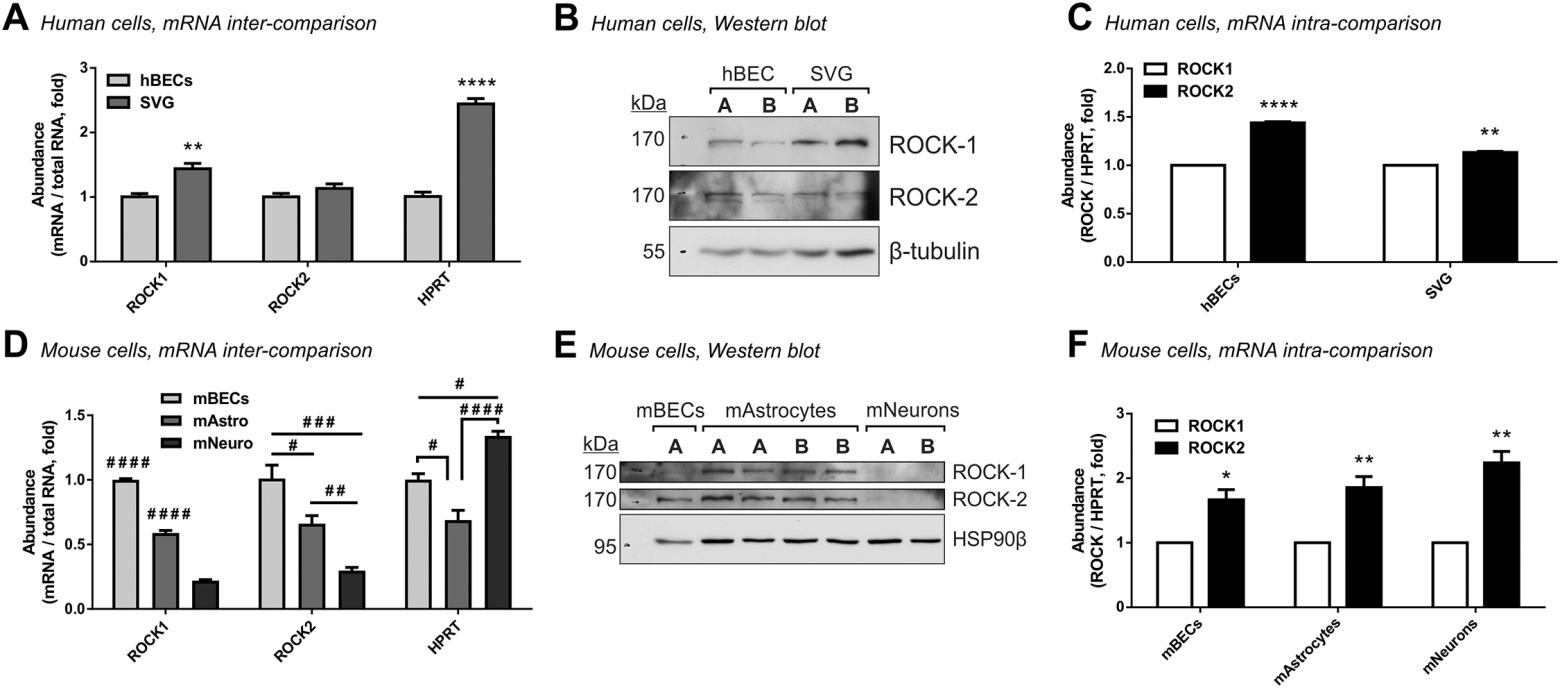
ROCK-2 is the primary isoform expressed in cells of the neurovascular unit. Real-time RT-PCR (**A**; n = 4 for each cell type) and western blot analysis **(B)** showing higher steady-state levels of ROCK-1 and similar levels of ROCK-2 (per equal quantities of total RNA and proteins) in unstimulated human SVG astrocytes compared to human brain microvascular endothelial cells (hBECs). **(C)** Comparative qPCR analysis (by ΔΔCt, normalising to HPRT) in hBECs and SVGs (n = 4 each) demonstrating that ROCK-2 is the principal ROCK transcript expressed in each human cell type. **(D)** Real-time RT-PCR in equal quantities of RNA from unstimulated primary mouse neurons (mNeuro; n = 4) and primary mouse astrocytes (mAstro; n = 4) relative to primary mouse brain endothelial cells (mBECs; n = 3). mBECs are enriched in ROCK-1 and ROCK-2 transcripts while mouse neurons contains the least. **(E)** Western blot analysis of ROCK-1 and ROCK-2 protein levels in unstimulated mouse BECs, astrocytes and neurons. **(F)** Comparative qPCR analysis of ROCK-1 *versus* ROCK-2 mRNA relative to HPRT within primary mouse BECs (n = 3), astrocytes (n = 4) and neurons (n = 4). The ROCK-2 isoform is dominant also within each of the mouse brain cell types. In all panels bars represent mean±SEM. *P<0.05, **P<0.01, ****P<0.0001 compared to the respective reference column (on the left) by two-tailed unpaired (A) or paired (B, D) t-tests. # *P*<0.05, ## *P*<0.01, ### *P*<0.001, #### *P*<0.0001 (compared to all other groups if unspecified) by one-way ANOVA with Tukey’s post hoc analysis. In panels (B) and (E) the letters A and B represent independent cultures.

ROCK-2, the main brain isoform of Rho-kinase [25, 26], has been primarily localised to neurons [25, 27] and non-vascular astrocytes [27, 28] rather than to blood vessels. Because the relevance of ROCK-2 to neurovascular function has not been extensively studied, we first looked at steady state levels of the two ROCK isoform transcripts in human and mouse brain cells which comprise the BBB, namely brain endothelial cells (BECs) and astrocytes, and further compared them to the abundance of ROCK mRNA in neurons. Based on total RNA, human immortalised astrocytes contained higher levels of ROCK-1 (1.44 ± 0.08 fold, *P*<0.01) and similar levels of ROCK-2 mRNA (1.13 ± 0.07 fold, not significant) compared to human BECs (Fig 1A). Western blot analysis confirmed these ratios of ROCKs also at the protein level (Fig 1B). Further comparison of the two ROCK mRNA isoforms within each cell type demonstrated that ROCK-2 is the more abundant isoform in both cells compared to ROCK-1 (1.44 ± 0.046 fold, *P*<0.0001 in human BECs and 1.136 ± 0.047 fold, *P*<0.01 in SVGs; Fig 1C). These data suggests that ROCK-2 may be a dominant player in both the endothelial and the astrocytic compartments of the human BBB.

A similar analysis in primary cultures of mouse BECs, astrocytes and neurons revealed that mouse BECs are in fact the richest in the two mRNA isoforms of ROCK and that mouse neurons surprisingly contain the least per total RNA (0.22 ± 0.008 fold for ROCK-1, *P*<0.0001 and 0.3 ± 0.025 fold for ROCK-2, *P*<0.001 compared to mouse BECs; Fig 1D). Mouse astrocytes held intermediate levels of ROCK-1 and ROCK-2 compared to mouse BECs (0.59 ± 0.02 fold for ROCK-1, *P*<0.0001 and 0.66 ± 0.06 fold for ROCK-2, *P*<0.05; Fig 1D). Importantly, despite their lower ROCK expression, mouse neurons contain the highest levels of the house-keeping gene HPRT, as expected from their high metabolic state (1.34 ± 0.037 fold compared to mouse BECs, *P*<0.05; Fig 1D), authenticating our observations with ROCK. On the protein level, ROCK-1 was predominantly expressed in mouse astrocytes and mildly in mouse BECs, while both cell types expressed ROCK-2 at comparable levels. The two ROCK proteins were notably undetectable in cultured neurons (Fig 1E) as predicted from our real-time PCR. Within each mouse primary cell type, ROCK-2 was the dominant isoform compared to ROCK-1, ranging from 1.66 ± 0.12 fold, *P*<0.05 in BECs, 1.84 ± 0.1 fold, *P*<0.01 in astrocytes and 2.25 ± 0.22 fold, *P*<0.01 in neurons (Fig 1F). Taken together, ROCK-2 emerges as a strong candidate to play central roles also in the mouse neurovascular unit, being highly expressed compared to ROCK-1 in all brain cell types and particularly enriched in rodent brain endothelial cells and glia compared to neurons.

**Fig 2:**
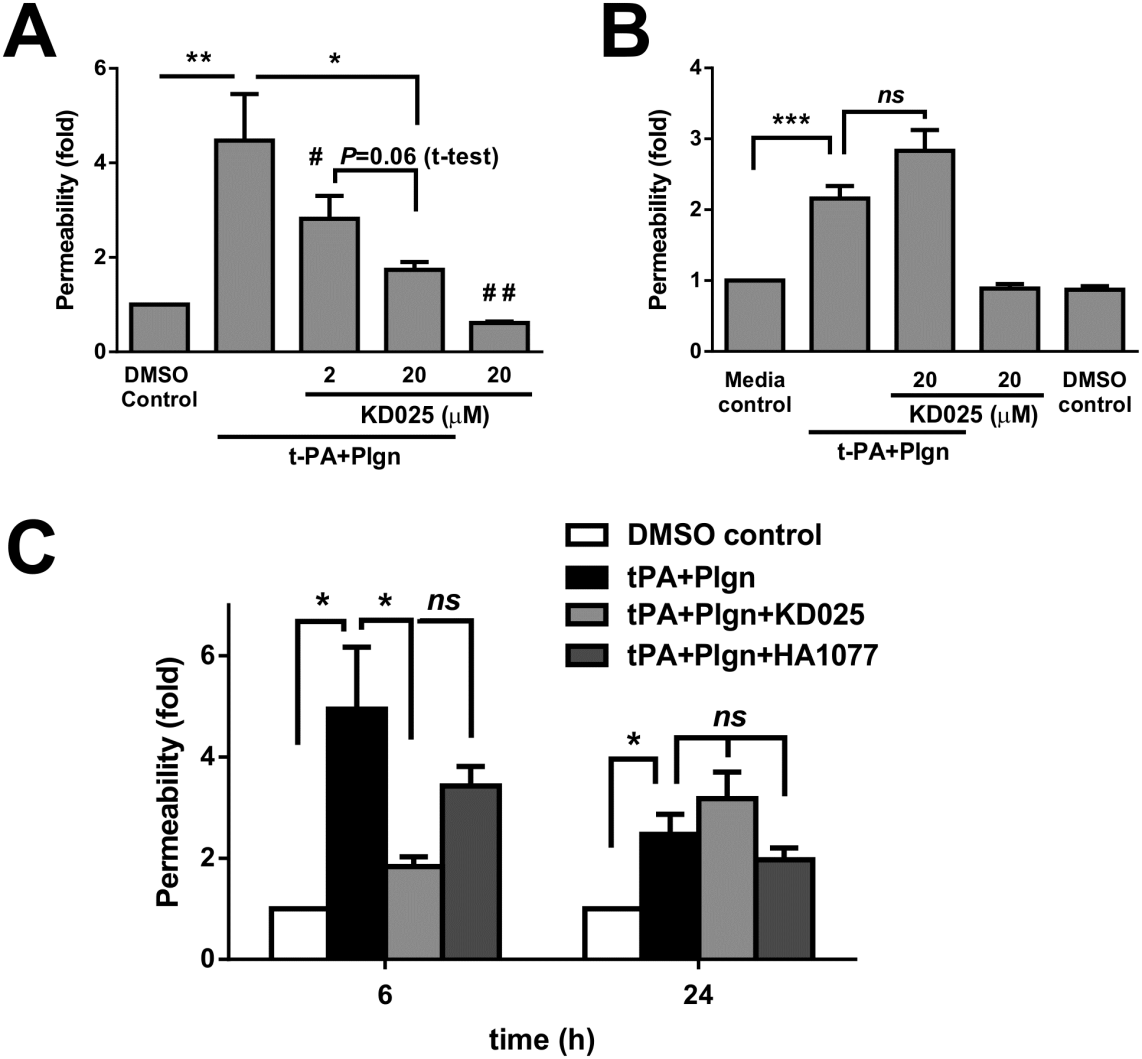
Selective ROCK-2 inhibition effectively blocks early, but not late rt-PA-induced increases in BBB permeability under normal conditions. **(A, B)** Permeability changes in the *in vitro* human BBB 6 h (**A**; n = 4) or 24 h (**B**; n = 4–10) post stimulation under normal conditions with DMSO as control or with rt-PA (25nM) and human plasminogen (plgn; 100nM), with or without KD025 (2 and 20μM, added to both luminal and abluminal chambers). **(C)** Comparison of selective ROCK-2 inhibition by KD025 (20μM) *versus* non-selective ROCK inhibition by fasudil (HA1077; 20μM) against rt-PA+plasminogen (25nM+100nM, respectively) 6 h and 24 h after treatment under normal conditions. KD025, but not HA1077, displays strong protective capacity at 6 h, but not at 24 h. n = 3–4. Bars represent mean±SEM. *P<0.05, **P<0.01, ***P<0.001 by one-way ANOVA with Tukey’s post hoc analysis. # *P*<0.05 compared to rt-PA+Plgn, ## *P*<0.01 compared to DMSO control and specified *P* values are by two-tailed paired t-test.

We previously linked the effects of t-PA and plasmin on the BBB to activation of the ROCK pathway in astrocytes [7, 20]. Having established the dominance of ROCK-2 in cells of the BBB, we next tested *in vitro* whether selective inhibition of ROCK-2 with the novel inhibitor KD025 has merit in BBB protection from these proteases. As shown in Fig 2A, KD025, co-applied together with rt-PA and plasminogen for 6 h in our BBB model, concentration-dependently blocked permeability increases under normal glucose and oxygen levels (hereafter referred to as “normoxia”), reaching a maximal blocking effect at 20μM (4.47 ± 0.98 fold with rt-PA and plasminogen *vs*. 1.74 ± 0.17 fold with KD025, *P*<0.05). Interestingly, KD025 applied alone also tightened the BBB compared to control conditions (0.61 ± 0.03 fold, *P*<0.01; Fig 2A), suggestive of a general benefit to barrier function by selective ROCK-2 inhibition. We next assessed the activity of KD025 under normoxia during prolonged rt-PA and plasminogen stimulation (24 h; Fig 2B) which surprisingly resulted in weaker permeability rises (2.16 ± 0.18 fold) than in 6 h (4.47 ± 0.98 fold; Fig 2A). Permeability analysis against an insert without cells (which is not influenced by time in culture) showed no differences in control inserts between 6 and 24 h, suggesting that the BBB recovers and closes as rt-PA stimulation prolongs (not shown). While KD025 on its own was well-tolerated up to 24 h, no benefit was observed against rt-PA and plasmin at this time-frame (Fig 2B). In fact, a detrimental trend for enhanced BBB permeability was identified with prolonged use of KD025 together with rt-PA (Fig 2B). In a sub-set set of these experiments we further compared under normoxic conditions selective ROCK-2 inhibition with KD025 to nonselective ROCK inhibition using fasudil (HA1077; 20μM) (Fig 2C). In comparison to KD025, which reduced rt-PA-mediated permeability changes by 79% at 6 h (4.94 ± 1.22 fold without *vs*. 1.83 ± 0.19 fold with KD025, *P*<0.05; Fig 2C), fasudil treatment resulted in a non-significant 38.3% reduction in permeability (down to 3.43 ± 0.38 fold; Fig 2C). At 24 h, both KD025 and HA1077 displayed no significant protection against rt-PA-induced BBB opening. The trends, however, were reversed, as fasudil still dampened rt-PA-mediated BBB opening by 20.2% while KD025 increased permeability at 24 h (Fig 2C). Overall, this investigation under normoxia fundamentally shows that selective ROCK-2 inhibition is not only effective but also a preferable strategy to tackle the impact of rt-PA and plasmin on the BBB within the initial hours post stimulation.

**Fig 3:**
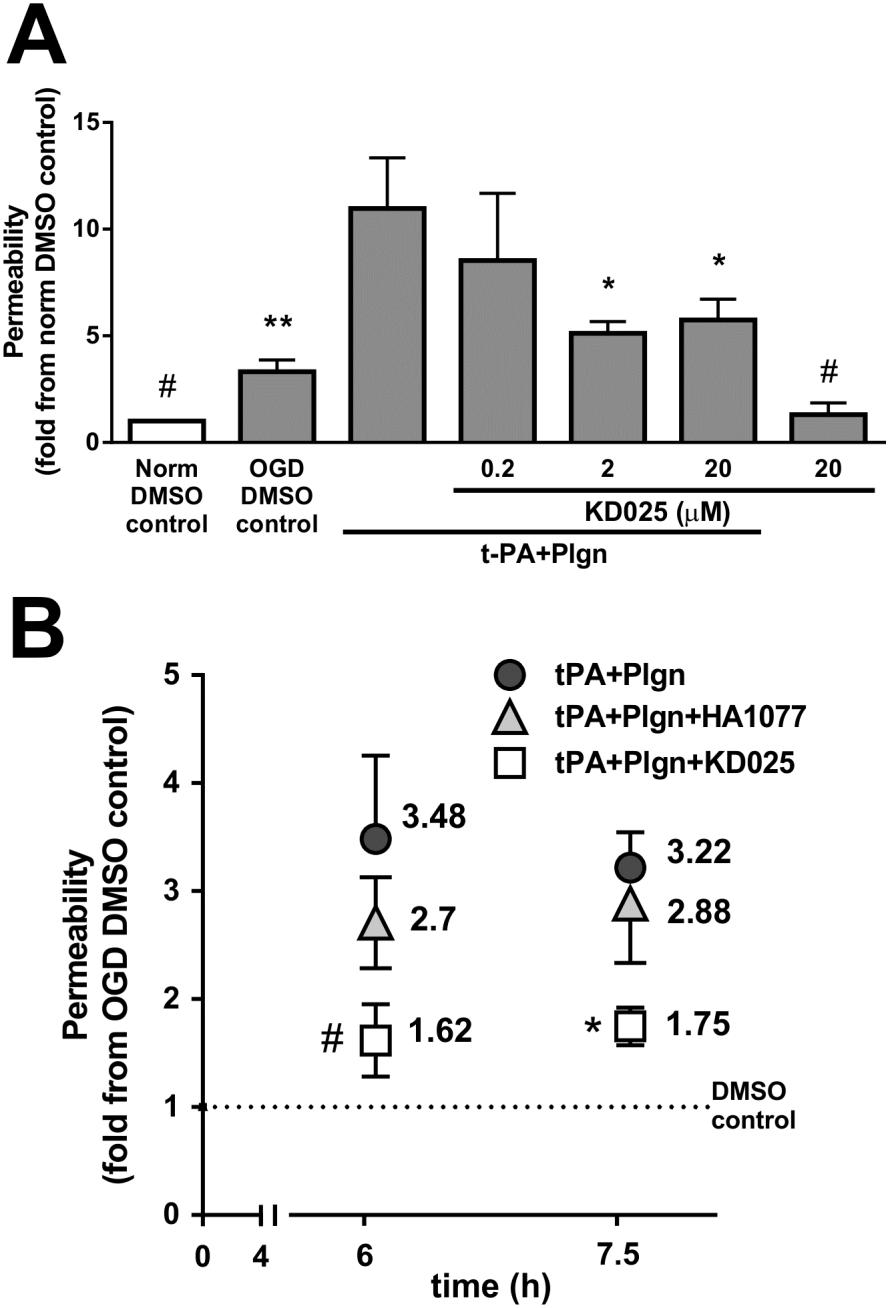
Selective ROCK-2 inhibition by KD025 blocks rt-PA-induced effects on BBB permeability under stroke-like conditions. **(A)** Permeability changes in the *in vitro* human BBB 7.5 h post stimulation under oxygen-glucose deprivation (OGD) with DMSO as control or with rt-PA (25nM) and human plasminogen (plgn; 100nM), with or without KD025 (0.2, 2 and 20μM, added to both luminal and abluminal chambers). Data is presented relative to DMSO control under normoxia. n = 3, Bars represent mean±SEM. *P<0.05, **P<0.01 compared to rt-PA+Plgn by one-way ANOVA with Newman–Keuls post hoc analysis. #*P*<0.05 compared to DMSO OGD control by two-tailed t-test. **(B)** Comparison of selective ROCK-2 inhibition by KD025 (20μM) *versus* non-selective ROCK inhibition by fasudil (HA1077; 20μM) against rt-PA+plasminogen (25nM+100nM, respectively) 6 h and 7.5 h after treatment under OGD (relative to DMSO control under OGD). n = 3 for 6 h, n = 4 for 7.5 h. Data points represent mean±SEM. *P<0.05 by one-way ANOVA with Tukey’s post hoc analysis. #*P*<0.05 compared to rt-PA+Plgn by one-tailed paired t-test.

Since t-PA-induced effects on the BBB are of particular relevance in the context of ischaemic stroke, it was crucial to assess selective ROCK-2 inhibition also under stroke-like conditions. Indeed, simultaneous addition of KD025 with rt-PA and plasminogen under oxygen-glucose deprivation for 7.5 h (a time-frame where OGD injury became apparent compared to normoxic control, *P*<0.05; Fig 3A) strongly protected the human BBB model in a concentration-dependent manner (Fig 3A), with most effective inhibition achieved already at 2μM (10.97 ± 2.37 fold with rt-PA+plasminogen *vs*. 5.11 ± 0.55 fold with KD025 at 2μM compared to normoxic control, *P*<0.05). As previously seen under normoxia (Fig 2A), KD025 applied alone tightened baseline permeability of the *in vitro* BBB also under OGD (*P*<0.05; Fig 3A). Lastly, when compared to nonselective ROCK inhibition with HA1077 under OGD, we observed effective inhibition of rt-PA+plasminogen-induced BBB opening only by KD025 (20μM) at both 6 h and 7.5 h (*P*<0.05; Fig 3B). Application of HA1077 (20μM) in the same experiments resulted in weak- to no inhibitory activity at 6 h and 7.5 h, respectively, indicative of a rapid loss of fasudil efficacy under OGD. Hence, selective ROCK-2 inhibition by KD025, in contrast to a nonselective ROCK inhibition strategy, has a potent capacity to antagonise the actions of rt-PA and plasmin on the BBB under stroke-like conditions.

**Fig 4:**
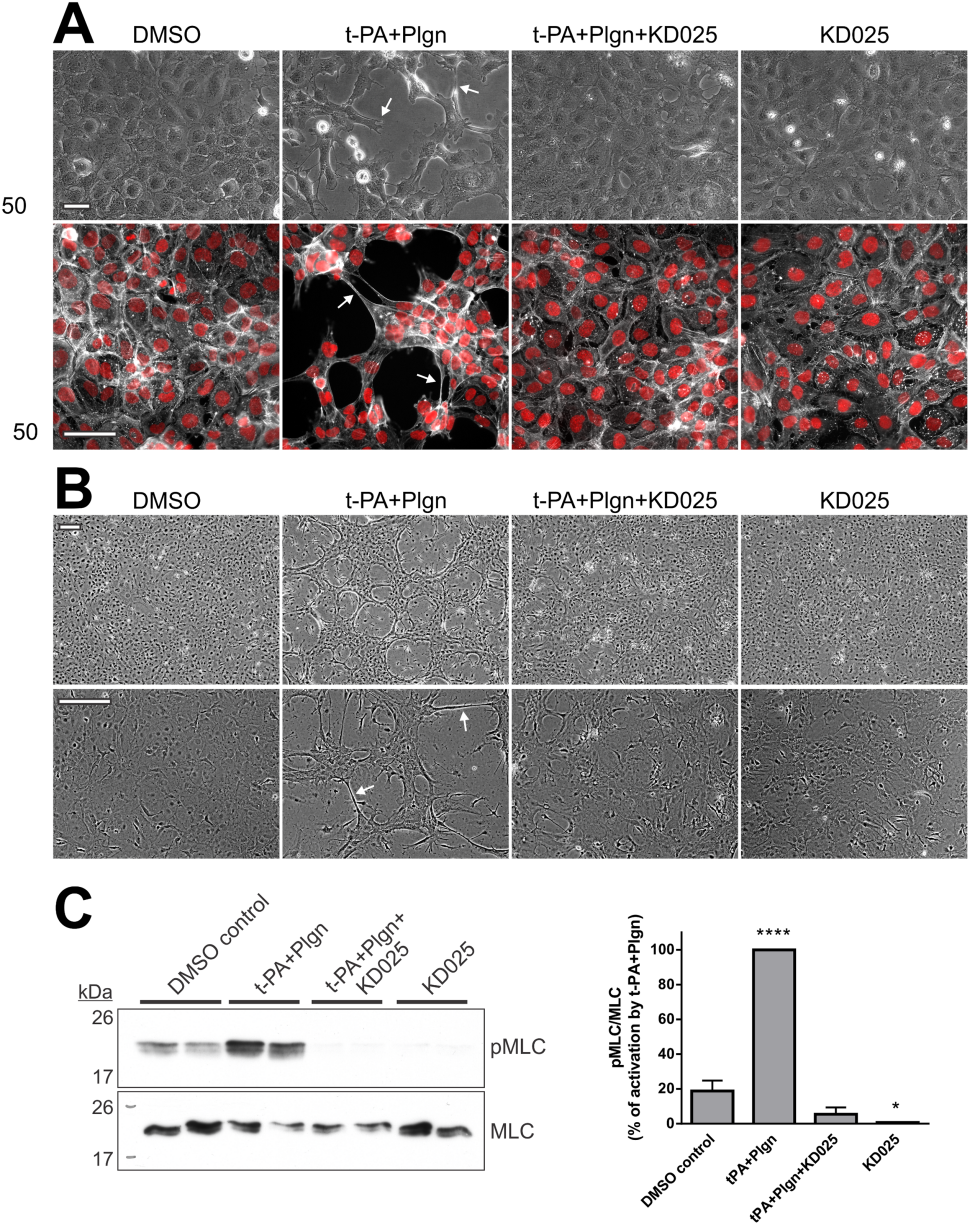
Selective ROCK-2 inhibition blocks rt-PA-induced morphological changes and ROCK signalling in human and mouse astrocytes. **(A)** Representative phase-contrast images (top panels) and double immunofluorescence images of the actin cytoskeleton (phalloidin; grey) and nuclei (Hoechst; red) (bottom panels) of SVG human astrocytes demonstrating blockade of morphology changes (arrows) by KD025 after 6 h treatment with DMSO (control) or with rt-PA+plasminogen (t-PA+Plgn; 25nM+100nM), in the presence or absence of KD025 (20μM). Scale bars = 50μm. **(B)** Representative phase-contrast images of primary mouse astrocytes treated overnight (13–16 h) as stipulated in A. n = 3. Scale bars = 200μm. Arrows depict changes in cell morphology. **(C)** Representative western blot analysis (left panel) and quantification (right panel) of phosphorylated myosin light chain levels (pMLC per total MLC) in primary mouse astrocytes treated for two hours with rt-PA+Plgn (50nM+100nM, respectively), with or without KD025 (20μM). Ratios obtained in the rt-PA+Plgn group were assigned a value of 100%. KD025 fully abolishes rt-PA- and plasmin-triggered MLC phosphorylation in mouse astrocytes. n = 3. Bars represent mean±SEM. ****P<0.0001 compared to all other groups, *P<0.05 compared to DMSO control by one-way ANOVA with Tukey’s post hoc analysis.

Mechanistically, we previously reported that fasudil protected the BBB by inhibition of rt-PA+plasminogen-triggered, ROCK-induced morphological changes and myosin light chain (MLC) phosphorylation in mouse and human astrocytes. Notably, fasudil did not influence similar cellular events in brain endothelial cells [20]. Because ROCK-2 is enriched in both brain endothelial cells and astrocytes (Fig 1), we tested the capacity of selective ROCK-2 inhibition to protect each of the cell types comprising our human BBB model. Of note, rt-PA-induced morphological changes in BECs and SVGs were not a consequence of cell-death and detachment as no LDH release or substantial reduction in cell viability (by the MTS assay) were evident up to 17 h post stimulation (interestingly, treatment with KD025 reduced baseline LDH levels in BECs at 17 h, indicating cyto-protection by selective ROCK-2 inhibition) (S2 Fig). In line with our previous report with fasudil [20], KD025 effectively reduced morphological alterations and preserved the actin cytoskeletal structure in human SVG cells 6 h post stimulation with rt-PA and plasminogen (Fig 4A), in correlation with the ability of KD025 to block permeability changes at this time frame (Fig 2A). The inhibitory effect of KD025 on SVG morphology was maintained up to 24 h post protease addition (not shown). KD025 further blocked morphology changes (Fig 4B) and MLC phosphorylation (*P*<0.0001; Fig 4C) in primary mouse astrocytes, indicating that selective inactivation of ROCK-2 has a similar capacity as nonselective inhibition strategy to reduce rt-PA and plasmin activation of astroglia.

**Fig 5:**
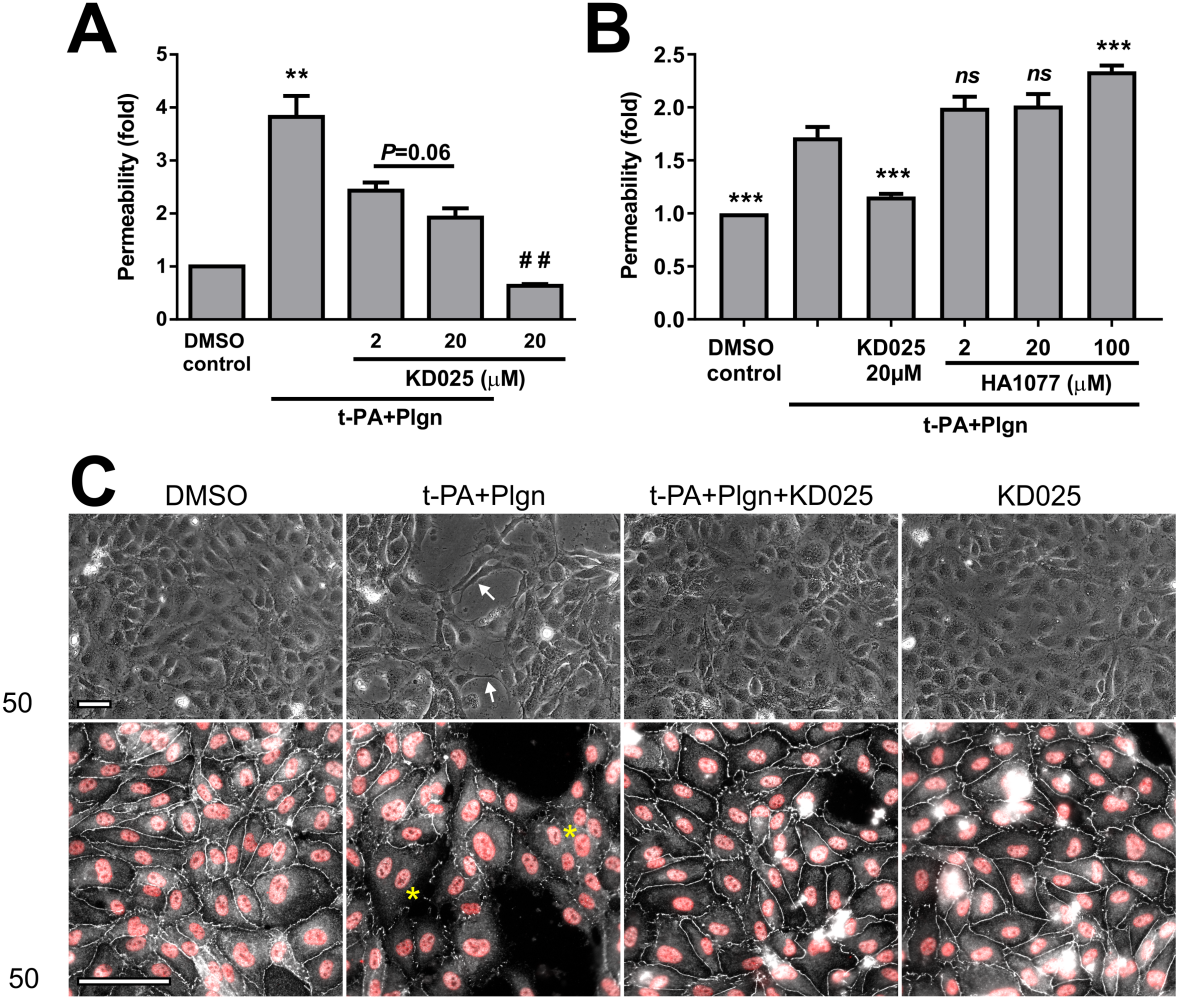
Only KD025, but not fasudil, protects brain endothelial cells from rt-PA and plasmin. **(A)** KD025 concentration-dependently blocks permeability changes in primary human brain microvascular endothelial cells (BECs; cultured without astrocytes) treated for 6 h with DMSO as control or with rt-PA (25nM) and human plasminogen (plgn; 100nM), with or without KD025 (2 and 20μM). n = 4. **(B)** Comparison of selective ROCK-2 inhibition by KD025 (20μM) *versus* non-selective ROCK inhibition by fasudil (HA1077; 2, 20 and 100μM) against rt-PA+plasminogen (25nM+100nM, respectively) in BECs, as assessed 6 h post stimulation. HA1077 displays no protective capacity in BECs. n = 3. Bars represent mean±SEM. **P<0.01 compared to all other groups, ***P<0.001 compared to t-PA+Plgn by one-way ANOVA with Tukey’s post hoc analysis. ## *P*<0.01 compared to DMSO control and *P* = 0.06 by two-tailed paired t-test. **(C)** Representative phase-contrast micrographs (top panels) and double immunofluorescence images of zonula occludens 1 (ZO-1, representing tight junctions (TJs); white) and nuclei (Hoechst; red) (bottom panels) of BECs stimulated for 6 h with DMSO (control) or with rt-PA+plasminogen (25nM+100nM, respectively), in the presence or absence of KD025 (20μM). KD025 attenuates morphological changes (arrows) and preserves TJs in endothelial cells. Asterisks depict areas with degraded TJs. Scale bars = 200μm.

Importantly, however, a major difference between the activity of KD025 and fasudil was revealed in human BECs. KD025 potently and concentration-dependently blocked permeability rises induced over 6 h by rt-PA and plasminogen in monolayers of BECs alone (3.82 ± 0.4 fold with rt-PA+ plasminogen *vs*. 1.92 ± 0.17 fold with KD025 at 20μM, *P*<0.01; Fig 5A) and tightened BEC permeability also under basal condition (without protease stimulation, *P*<0.01; Fig 5A). In contrast, fasudil did not reduce rt-PA and plasminogen effects in BECs alone at a wide concentration range (2–100 μM) and in fact increased BBB permeability at the top concentration compared to a protective action of KD025 (Fig 5B). Treatment with KD025 further prevented morphological changes and tight junction breakdown (observed as fragmentation and disappearance of ZO-1 staining along cell-cell boundaries) which developed in BECs 6 h post stimulation with rt-PA and plasminogen (Fig 5C). These effects on BEC permeability and morphology could not be achieved with fasudil before [20]. Yet, in line with its inability to block permeability changes 24 h post treatment (Fig 2), KD025 did not prevent morphological alterations in BECs during overnight stimulation (not shown), Taken together, our results identify ROCK-2 as central to the acute effects of rt-PA and plasmin in both BECs and astrocytes and importantly suggest that *selective* ROCK-2 inhibition (which preserves ROCK-1 activity) is specifically required to antagonise rt-PA-induced detrimental responses in the brain endothelium.

**Fig 6:**
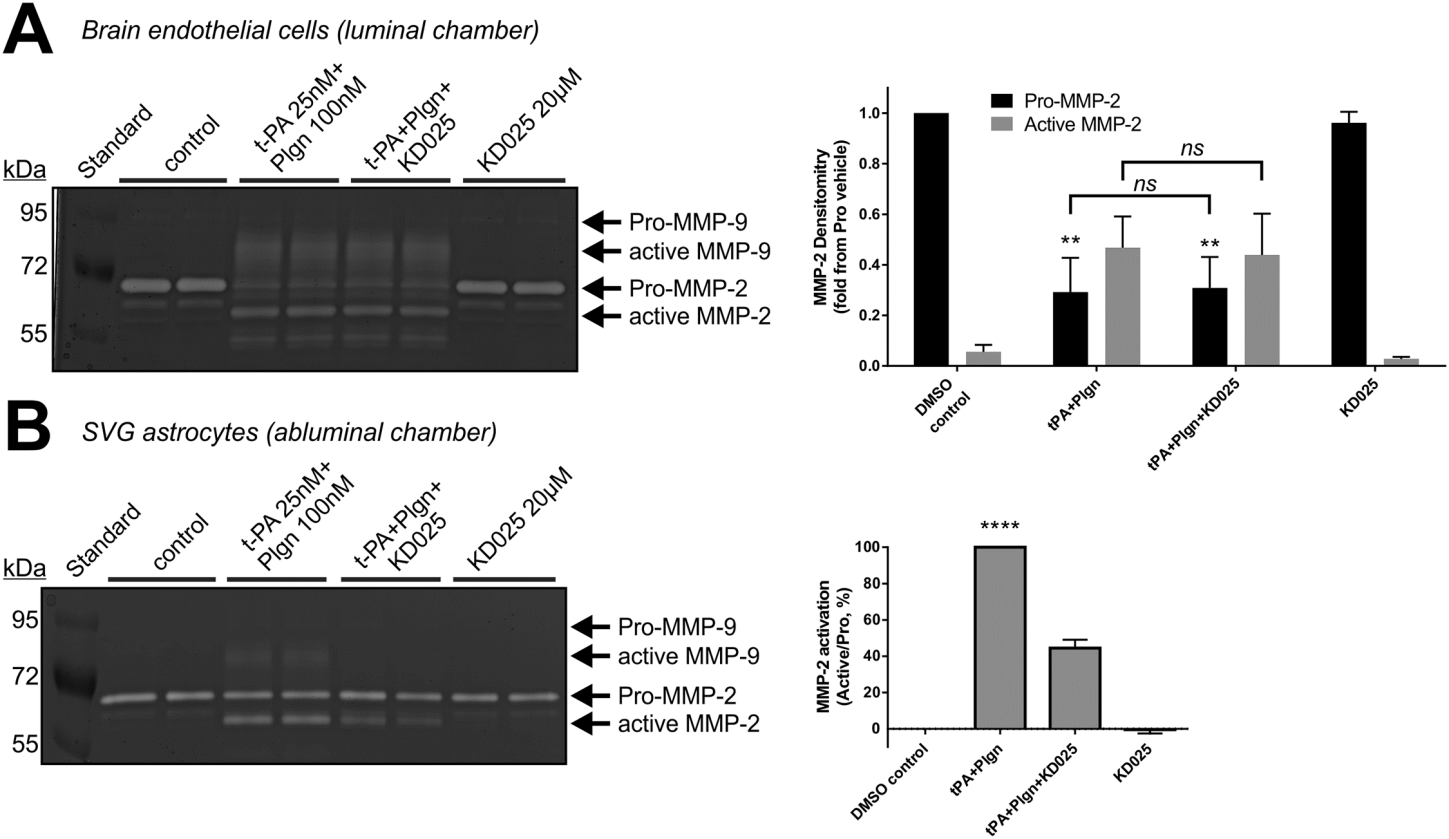
Selective ROCK-2 inhibition blocks rt-PA-induced MMP-2 activation in SVG human astrocytes, but not in BECs. Representative gelatine zymogram (left panels) and quantitation by densitometry (right panels) of active MMP-2, pro-MMP-2 (**A**) or their ratio (**B**) in media harvested from the luminal (endothelial; **A**) or the abluminal (astrocytic; **B**) chambers of the *in vitro* BBB 6 h post stimulation with DMSO (control) or with rt-PA+plasminogen (t-PA+Plgn; 25nM+100nM, respectively), in the presence or absence of KD025 (20μM). In (B) ratios obtained in the t-PA+Plgn group were assigned a value of 100%. KD025 reduces MMP activation in SVG, but not in BECs. n = 4. ****P<0.0001 against all other groups by one-way ANOVA, **P<0.01 against control or KD025 by two-way ANOVA with Tukey’s post hoc analysis.

We finally tested the effect of selective ROCK-2 inhibition on matrix-metalloproteases (MMP) production and activation in our BBB model under normoxia since MMPs, heavily linked to BBB disruption by rt-PA [8], were reported to be regulated *in vitro* by ROCK downstream of t-PA in both brain endothelial cells [22] and astrocytes [21]. Our gelatine zymograms of conditioned medium taken 6 h post stimulation from the endothelial chamber showed significant pro-MMP-2 consumption (*P*<0.01) and MMP-2 and -9 activation by rt-PA and plasmin, which were not attenuated by KD025 (Fig 6A). In contrast, selective ROCK-2 inhibition decreased MMP-2 activation in human SVG astrocytes by 55%, (*P*<0.0001; Fig 6B), as previously reported with fasudil [21].

Similar observations were seen under OGD, rendering these effects on MMPs relevant for stroke (S3 Fig). Despite the influence of KD025 on astrocytic MMP activation, it is worth mentioning that utilisation of the broad spectrum MMP inhibitor GM6001 (10μg/ml) together with rt-PA and plasminogen induced only a small yet significant reduction (*P*<0.05) in human BBB permeability and did not block rt-PA-induced morphological changes in mouse astrocytes (S4 Fig). Overall, these data suggest that selective ROCK-2 inhibition interferes with t-PA/plasmin-induced MMP activation in astrocytes, but not in BECs, but also indicate that MMPs play only a partial role in t-PA- and plasmin-mediated BBB disruption in this *in vitro* human model.

## Discussion

In this study we harnessed the improved specificity of KD025, a newly available selective ROCK-2 inhibitor [15, 38, 40], to identify that ROCK-2 is the predominant isoform engaged by rt-PA in brain endothelial cells and astrocytes to cause impairment of the BBB. This important finding refines previous observations where nonselective inhibition of ROCK protected the BBB [20, 22, 24], but not isolated BECs [20], from rt-PA. Our study therefore suggests that selective inhibition of ROCK-2 is more suitable for BBB protection during rt-PA treatment and that simultaneous inhibition of both ROCK isoforms is sub-optimal for this purpose.

Such mechanistic refinement might be a crucial factor for translation; indeed, the vast clinical experience and good safety profile demonstrated with fasudil, a nonselective ROCK inhibitor already in clinical use [11, 12], makes its simultaneous utilisation with rt-PA a feasible way forward to protect the BBB during thrombolysis. Yet, fasudil was tested successfully in human stroke only in the non-acute phase of AIS, with mean time from onset to treatment of ~24 h [19]. In this practice, fasudil was mainly aiming at secondary injury mechanisms after stroke, such as endothelial inflammation and hemodynamic dysfunctions [19]. Translational implementation of a pan ROCK inhibitor also in the acute settings of AIS alongside rt-PA, as performed in rodents [22, 24], may face limitations due to potent systemic effects of nonselective ROCK inhibitors, such as vasodilation and hypotension, which could lead to unwanted brain hypo-perfusion and critical exacerbation of ischaemic injury [41]. Shin *et al*. [41], which found evidence for differing mechanisms of ROCK inhibitor-mediated vasodilation between the systemic and cerebral circulation, therefore called for a more selective ROCK inhibition strategy in stroke. The opportunity to selectively inhibit ROCK-2 by KD025 may provide a plausible solution, as KD025 was shown to retain the beneficial consequences of ROCK inhibition in rodent stroke without significant hypotensive issues [15]. Together with our results showing for the first time the additional ability of KD025 to protect the BBB from rt-PA and plasmin, KD025 emerges as a novel candidate to effectively reduce detrimental off target effects of rt-PA during thrombolysis with minimal systemic concerns.

The contribution of ROCK-2 to BBB function and dysfunction only now begins to unravel [45], owing to emergence of highly specific ROCK inhibitors. This relationship should not be taken for granted, as brain localisation studies commonly demonstrate ROCK-2 expression in neurons [25, 27] as well as in reactive astrocytes [27, 28], without marked immunoreactivity in cerebral blood vessels and in BECs. Nevertheless, a wealth of data strongly links brain endothelial ROCK activity to BBB breakdown during stroke. For example, elevated levels of phosphorylated myosin were detected in the ischaemic brain vessel wall [13, 46], indicative of universal vascular ROCK activity in these conditions; further, it is now well-accepted that the RhoA/ROCK pathway inversely regulates endothelial nitric oxide synthase (eNOS) and that the beneficial activity of ROCK inhibitors is largely mediated via upregulation and activation of eNOS [47], also shown in the brain during stroke [16, 41]; finally, in isolated BECs nonselective ROCK inhibition attenuated OGD-invoked oxidative stress [14], tight junction degradation [48, 49], barrier dysfunction [48–50] and rt-PA-induced cell death and MMP-9 upregulation [22], indicative of substantial participation of ROCKs in the ischaemic BEC response. A more specific ROCK-2 expression and/or function has been documented in endothelial cells of the umbilical cord [31, 36], lung [31, 33, 40], pancreas [32, 34] and recently in brain arterioles [45], where it plays distinct roles in proinflammatory cell adhesion molecule expression [36], maintenance and regulation of vascular permeability [31] and vascular tone [45], temporal MLC phosphorylation [33] and angiogenesis [32, 34]. Taken together, it could be speculated that ROCK-2 is most likely involved in regulation of the cerebral microcirculation which forms the BBB, particularly during pathological processes. Our study conclusively supports this notion; first, using real-time PCR and western blot analysis not only we confirmed the ROCK-2 transcript as the major ROCK isoform expressed in neurons and astrocytes, in line with the literature [25–28], but further demonstrated a marked and undescribed enrichment of both ROCK transcripts in brain endothelial cells and astroglia compared to neurons, with clear dominance of ROCK-2 compared to ROCK-1 also in the cerebral endothelial compartment. Thus, the major cellular constituents of the BBB (i.e. BECs and astrocytes) robustly express ROCK-2, suggestive of its importance to BBB function. Second, selective ROCK-2 inhibition significantly reduced rt-PA- and plasminogen- triggered permeability increases in BECs as well as in the *in vitro* BBB. To our knowledge, this is the first direct evidence of a fundamental role played by ROCK-2 in any BBB breakdown paradigm and in particular of its key function in brain endothelial damage and BBB disruption by rt-PA and plasmin.

Our study also shows better attenuation of rt-PA-mediated BBB impairment by selective ROCK-2 inhibition than pan ROCK inhibition, but only within the early hours post-stimulation (6–7.5 h) and notably under OGD (Figs 2 and 3). Importantly, despite the theoretical capacity of fasudil to target ROCK-2, only KD025, but not fasudil, was able to block rt-PA-triggered changes in permeability, cell morphology and tight junction structure in isolated BECs (Fig 5). Yet in astrocytes, both selective and nonselective ROCK inhibition induced similar protective effects (Fig 4 and reference [20]). These curious findings highlight the brain endothelium as responsible for the improved efficacy of KD025 compared to fasudil in our BBB model. Furthermore, the data intriguingly suggests differential and time-dependent roles of the two ROCK isoforms in BECs (and therefore at the BBB) downstream of rt-PA, with ROCK-2 being a major contributor to BBB impairment at early time frames.

Comparable observations by Beckers and colleagues in human lung and umbilical cord endothelial cells [31] support various aspect of these conclusions. Indeed, siRNA-induced silencing of ROCK-2, but not ROCK-1, significantly reduced thrombin-mediated permeability increases both *in vitro* and *in* vivo [31]. ROCK-2 was similarly involved in rapid permeability rises post thrombin treatment (within 1 h) while ROCK-1 became more prominent at a later stage [31]. Since plasmin was suggested to damage the BBB, at least in part, via cleavage of the thrombin receptor protease-activated receptor (PAR)-1 on BECs and astrocytes [7, 51] (a concept recently challenged *in vivo* [52]), it is tempting to speculate that the two studies might be elegantly linked via this common receptor. Moreover, poorer PAR-1 activation capacity of plasmin as well as altered PAR-1 cleavage sites compared to thrombin [7] could induce slower ROCK-2 upregulation in our BBB model, resulting in the wider time frames for KD025-mediated BBB protection against rt-PA and plasmin seen in our study (up to 7.5 h).

Additional mechanistic dissection by Beckers *et al*. suggested that KD025 exerts its protective actions by overall improvement of ROCK-2-affected basal endothelial permeability (rather than direct antagonism of the thrombin effect) and that the two ROCK isoforms play opposite roles mainly in the context of basal barrier function [31]. Similarly, we observed tightening of basal BBB and BEC permeability by selective ROCK-2 blockade under both normoxic and OGD conditions (Figs 2, 3 and 5). This effect of KD025 can generally increase the basic resistance of the BBB to rt-PA, but it also supports the concept that the two ROCK isoforms may perform opposite actions in brain endothelial cells.

Based on these findings, an integrated model can be put forward where simultaneous utilisation of KD025 with rt-PA may rapidly improve basal barrier function via ‘tightening’ of BEC permeability. In parallel, early detrimental ROCK-2 activity triggered by rt-PA in BECs and astrocytes is eliminated by KD025. The improved selectivity of KD025 may also allow conservation of ROCK-1 activity in the brain endothelium, producing a potent BBB protective action, yet beneficial activities of ROCK-1 require further experimental support. A second wave of ROCK inhibition may be required since sICH can develop within 36 h post rt-PA [2]. However a pan ROCK inhibitor, or optimally a ROCK-1-specific inhibitor, could be ideal against delayed effects of thrombolysis. Clearly, additional studies using isoform knockdown and ROCK-1-specific inhibitors are required to elucidate the exact temporal contribution of each ROCK isoform in this paradigm.

Importantly, the BBB is a complex structure and the cumulative effect of an inhibitor on all of its cellular components, for example in astrocytes and pericytes in addition to BECs, will determine the net benefit of the inhibition strategy during thrombolysis. While other approaches to counteract rt-PA involve a sole compartment (for example blockade of the astrocytic platelet-derived growth factor receptor α by Imatinib [53]), ROCK-2 might become an attractive unifying target for this purpose. In astrocytes, for instance, both KD025 (Fig 4) and fasudil [20] diminished t-PA- and plasmin-induced pMLC signalling and morphological changes (suggestive of unidirectional actions of ROCK-1 and ROCK-2 in astroglia), but selective ROCK-2 inhibition was sufficient to diffuse most of the rt-PA negative effect. Pericytes, belonging to the vascular smooth muscle cell (VSMC) lineage, may also benefit from ROCK-2 inhibition since rt-PA and plasmin were reported to influence vascular tone [7], an effect classically liked to ROCK activity in VSMC [10, 12]. Importantly, opposing effects of the ROCK isoforms on VSMC morphology were described, with predominant role of ROCK-2 in VSMC contractility [37]. Hence, selective ROCK-2 inhibition may theoretically be ideal also for antagonism of pericyte-triggered vasoconstriction of brain vessels during stroke. Additional studies will need to uncover cell-specific regulation of ROCKs at the BBB after rt-PA stimulation. Yet, fundamental benefits of selective ROCK-2 inhibition seem to span most compartments of the BBB and may therefore represent an optimal approach to shield the BBB during thrombolysis.

In summary, we have shown *in vitro* that ROCK-2 is the dominant isoform in cells of the BBB, playing an important role in barrier function loss during stroke-like conditions and particularly contributing to BBB impairment by rt-PA and plasmin. The use of a selective ROCK-2 inhibitor such as KD025 together with rt-PA strengthens *all* cellular layers of the BBB (unlike nonselective ROCK inhibitors which are less efficient in BECs) and may therefore effectively reduce sICH with minimal hypotensive side effects. Since intravenous thrombolysis with rt-PA is likely to remain a mainstay in AIS owing to its relative simplicity and proven cost *versus* benefit profile [1], such an outcome may greatly impact the limited number of treated stroke patients.

## Supporting information

S1 Fig ROCK-1 and ROCK-2 gene expression assays display similar efficiencies. To calculate qPCR reaction efficiency for human and mouse ROCK-1 and -2 gene expression assays, a dilution series (1 in 3) was made from human (SVG) and mouse (neurons) cDNA samples. The measured cycle threshold (Ct) values were plotted against the log of the relative cDNA dilution to establish a standard curve that permits the calculation of the efficiency (E) from the slope using the formula E = 10-1/Slope. Ideally, E values ~2 are considered efficient. **(A)** Standard curves of human ROCK-1 and ROCK-2 gene expression assays (Applied Biosystems assays Hs01127699_m1 and Hs00178154_m1, respectively).
Human ROCK-1 standard curve Y = (-3.296)X + 24.86. R2 = 0.9988. E = 2.010892.
Human ROCK-2 standard curve Y = Y = (-3.301)X + 24.49. R2 = 0.9991. E = 2.008808.
**(B)** Standard curves of mouse ROCK-1 and ROCK-2 gene expression assays (Applied Biosystems assays Mm00485745_m1 and Mm01270843_m1, respectively).
Mouse ROCK-1 standard curve Y = (-3.233)X + 25.84. R2 = 0.9946. E = 2.038363.
Mouse ROCK-2 standard curve Y = (-3.472)X + 26.19. R2 = 0.9983. E = 1.941005.
(TIF)

S2 Fig rt-PA, plasminogen and KD025 are not acutely cytotoxic to BECs and SVG astrocytes. BECs (**A**, **B**) and SVG astrocytes (**C**, **D**) were treated for 6 h or 17 h under serum-free conditions with rt-PA (t-PA; 25nM) and plasminogen (Plgn; 100nM), in the presence or absence of KD025 (20μM). Cell-death and cell viability were then determined using the lactate dehydrogenase (LDH) assay (**A**, **C**) and the methyl-thiazole-tetrazolium (MTS) assay (**B**, **D**), with total cell lysis and etoposide (100μg/ml) serving as positive controls, respectively (see Methods). No cell death or reduction in cell viability were detected 6 h post treatment with t-PA+Plgn and only mild reduction in viability was observed at 17 h. KD025 reduced LDH release from BECs at 17 h, indicative of a cyto-protective effect. n = 3–4. *P<0.05, **P<0.01 against control and specified *P* values in (A) by one-way ANOVA with Tukey’s post hoc analysis. #*P*<0.05, ##*P*<0.01, ###*P*<0.001 against control by two-tailed student t-test.
(TIF)

S3 Fig Selective ROCK-2 inhibition by KD025 attenuates rt-PA+Plasminogen-induced MMP activation in human immortalised astrocytes, but not in BECs, under oxygen-glucose deprivation (OGD). Gelatine zymograms of conditioned medium from the endothelial chamber (top panel) and the astrocytic chamber (bottom panel) taken 7.5 h post stimulation under OGD with rt-PA (25nM) and plasminogen (100nM), in the presence or absence of KD025 (20μM). Medium from cells under normoxia is included as reference (left lane). KD025 displays similar activity towards MMPs under OGD as under normoxia (see main article, Fig 6).
(TIF)

S4 Fig MMPs do not play a major role in rt-PA- and plasminogen-mediated permeability changes and in morphology alteration of mouse astrocytes. **(A)** rt-PA (t-PA; 25nM) and plasminogen (plgn; 50nM) were added to the luminal chamber of the *in vitro* human BBB either alone or together with the broad-spectrum MMP inhibitor GM6001 (10μg/ml), and permeability was assessed 24h later. GM6001 had only a modest (but significant) inhibitory effect on permeability increases induced by rt-PA and plasminogen. This suggests that MMPs have only a partial role in this phenomenon. n = 3. Bars represent mean±SEM. Statistical analysis by one way ANOVA with Newman-Keuls post hoc. **(B)** Representative phase-contrast images of primary mouse astrocytes 24h after treatment with rt-PA (50nM) + plasminogen (40nM) without or with GM6001 (10μg/ml). GM6001 was inert on its own and did not attenuate rt-PA /plgn-induced shape changes of mouse astrocytes, suggesting that this effect is MMP-independent. n = 2. Scale bars represent 100μm and 40μm in the X10 and X40 images, respectively. **(C)** GM6001 (10μg/ml), supplemented into the developing buffer of a gelatine zymography, effectively blocks active MMP-2 in conditioned medium of mouse astrocytes as well as MMP-2 and MMP-9 produced by HT1080 human fibrosarcoma cells.
(TIF)
