# Supplementary figures and images for: Selective inhibition of brain endothelial Rho-kinase-2 provides optimal protection of an in vitro blood-brain barrier from tissue-type plasminogen activator and plasmin
Source: PLoS One. 2017 May 16;12(5):e0177332. doi: 10.1371/journal.pone.0177332 (PMC5433693; doi:10.1371/journal.pone.0177332)

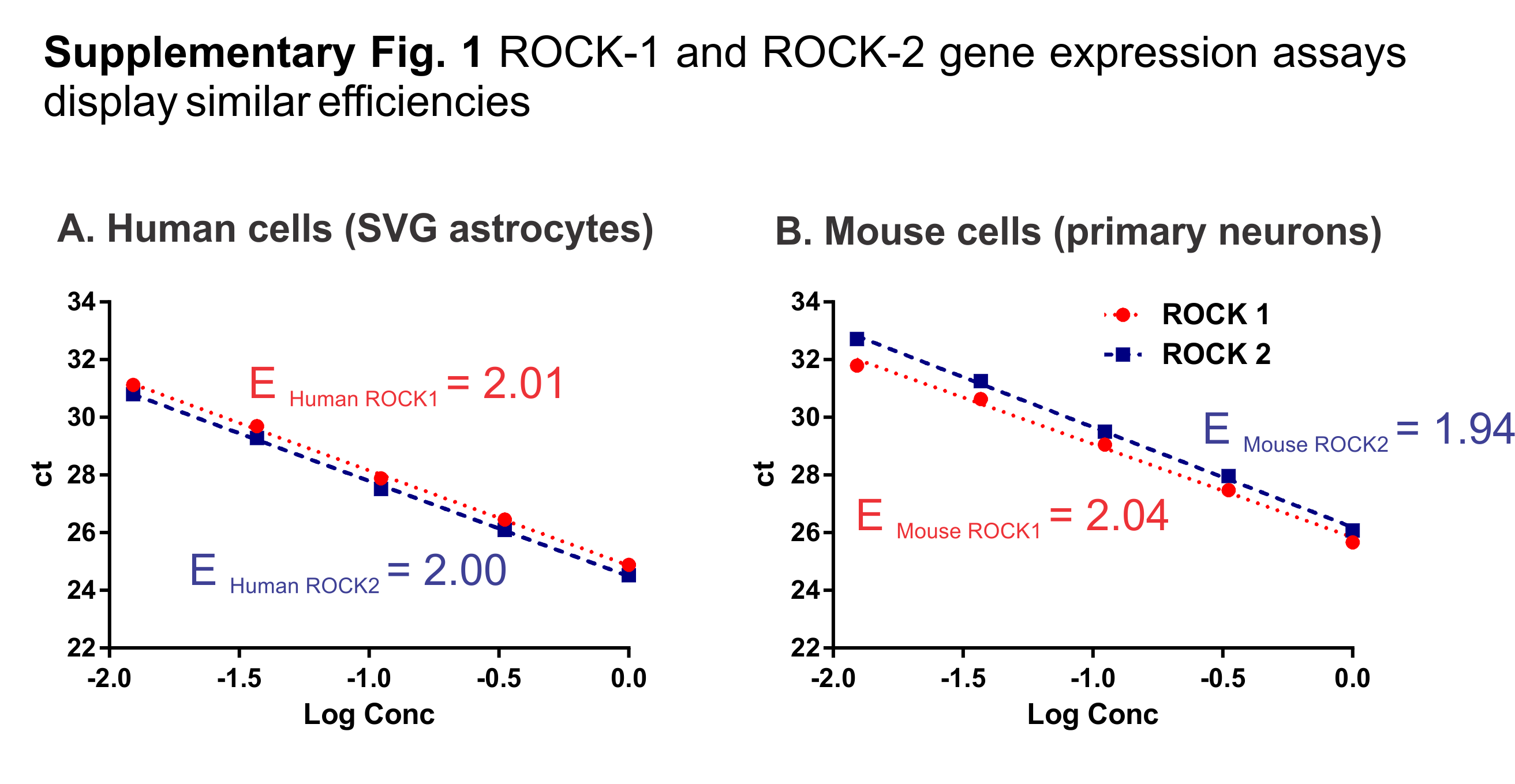

Supplement: S1 Fig — To calculate qPCR reaction efficiency for human and mouse ROCK-1 and -2 gene expression assays, a dilution series (1 in 3) was made from human (SVG) and mouse (neurons) cDNA samples. The measured cycle threshold (Ct) values were plotted against the log of the relative cDNA dilution to establish a standard curve that permits the calculation of the efficiency (E) from the slope using the formula E = 10-1/Slope. Ideally, E values ~2 are considered efficient. (A) Standard curves of human ROCK-1 and ROCK-2 gene expression assays (Applied Biosystems assays Hs01127699_m1 and Hs00178154_m1, respectively). Human ROCK-1 standard curve Y = (-3.296)X + 24.86. R2 = 0.9988. E = 2.010892. Human ROCK-2 standard curve Y = Y = (-3.301)X + 24.49. R2 = 0.9991. E = 2.008808. (B) Standard curves of mouse ROCK-1 and ROCK-2 gene expression assays (Applied Biosystems assays Mm00485745_m1 and Mm01270843_m1, respectively). Mouse ROCK-1 standard curve Y = (-3.233)X + 25.84. R2 = 0.9946. E = 2.038363. Mouse ROCK-2 standard curve Y = (-3.472)X + 26.19. R2 = 0.9983. E = 1.941005. (TIF) [file pone.0177332.s001.tif]

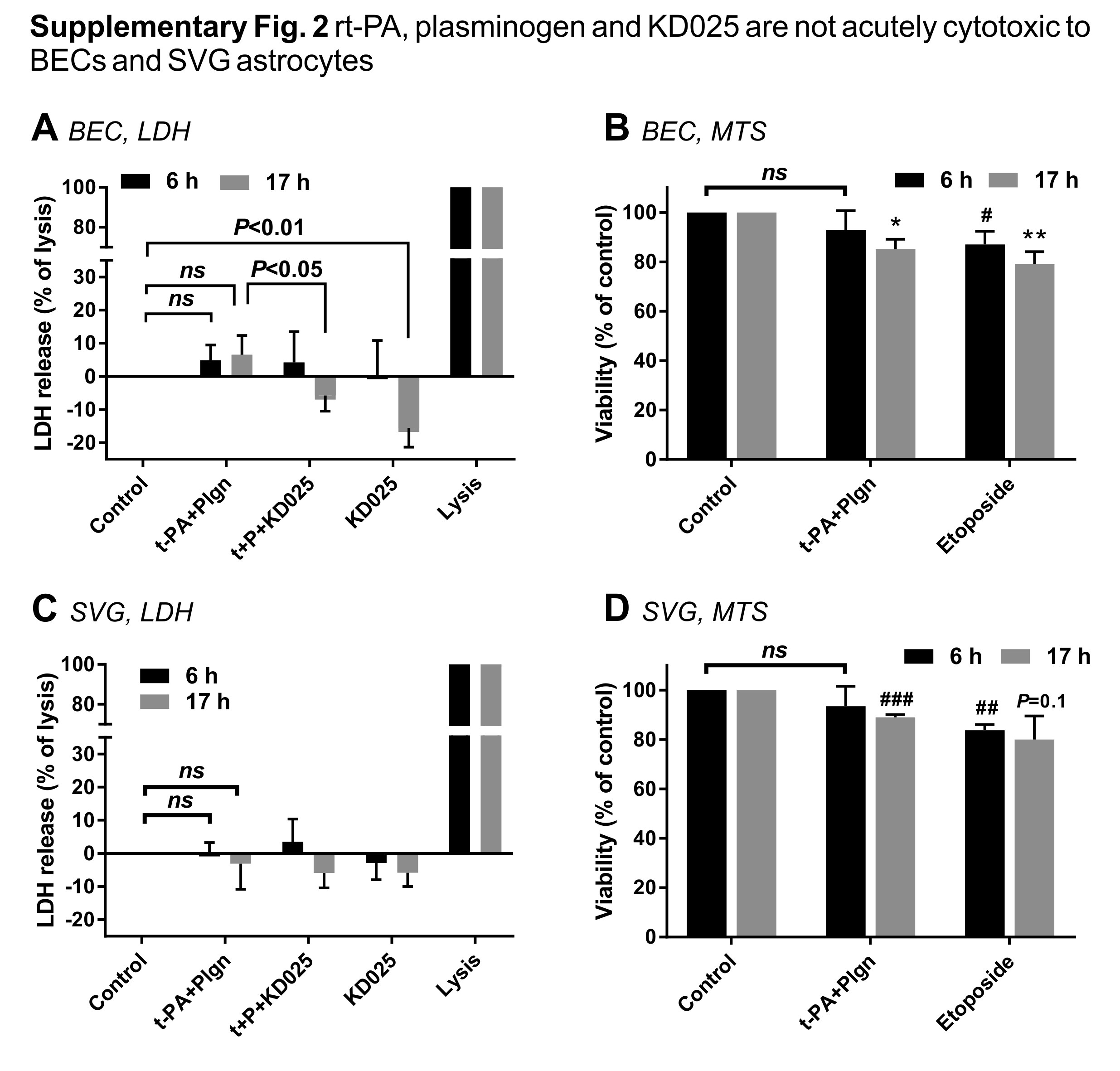

Supplement: S2 Fig — BECs (A, B) and SVG astrocytes (C, D) were treated for 6 h or 17 h under serum-free conditions with rt-PA (t-PA; 25nM) and plasminogen (Plgn; 100nM), in the presence or absence of KD025 (20μM). Cell-death and cell viability were then determined using the lactate dehydrogenase (LDH) assay (A, C) and the methyl-thiazole-tetrazolium (MTS) assay (B, D), with total cell lysis and etoposide (100μg/ml) serving as positive controls, respectively (see Methods). No cell death or reduction in cell viability were detected 6 h post treatment with t-PA+Plgn and only mild reduction in viability was observed at 17 h. KD025 reduced LDH release from BECs at 17 h, indicative of a cyto-protective effect. n = 3–4. *P<0.05, **P<0.01 against control and specified P values in (A) by one-way ANOVA with Tukey’s post hoc analysis. #P<0.05, ##P<0.01, ###P<0.001 against control by two-tailed student t-test. (TIF) [file pone.0177332.s002.tif]

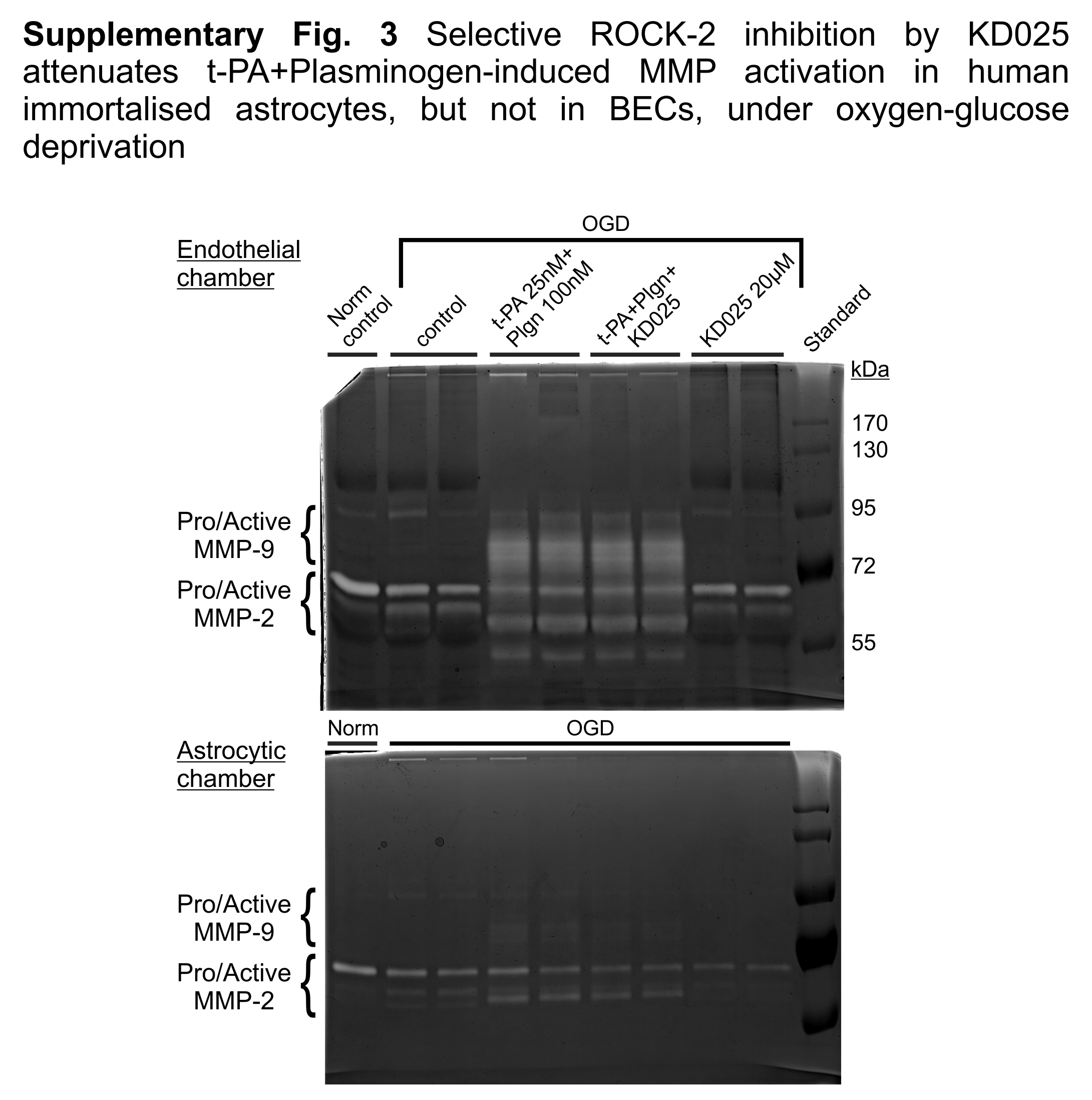

Supplement: S3 Fig — Gelatine zymograms of conditioned medium from the endothelial chamber (top panel) and the astrocytic chamber (bottom panel) taken 7.5 h post stimulation under OGD with rt-PA (25nM) and plasminogen (100nM), in the presence or absence of KD025 (20μM). Medium from cells under normoxia is included as reference (left lane). KD025 displays similar activity towards MMPs under OGD as under normoxia (see main article, Fig 6). (TIF) [file pone.0177332.s003.tif]

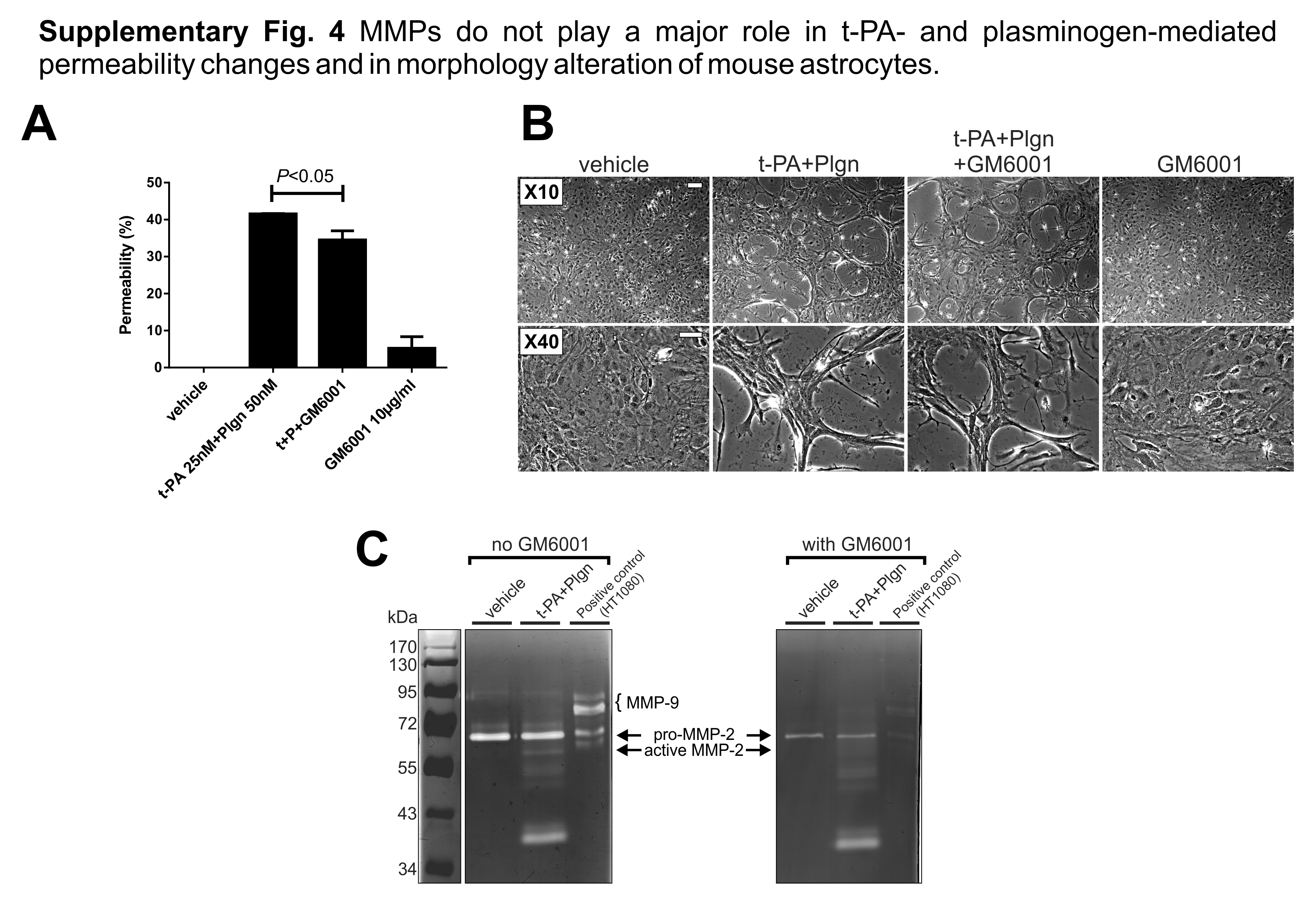

Supplement: S4 Fig — (A) rt-PA (t-PA; 25nM) and plasminogen (plgn; 50nM) were added to the luminal chamber of the in vitro human BBB either alone or together with the broad-spectrum MMP inhibitor GM6001 (10μg/ml), and permeability was assessed 24h later. GM6001 had only a modest (but significant) inhibitory effect on permeability increases induced by rt-PA and plasminogen. This suggests that MMPs have only a partial role in this phenomenon. n = 3. Bars represent mean±SEM. Statistical analysis by one way ANOVA with Newman-Keuls post hoc. (B) Representative phase-contrast images of primary mouse astrocytes 24h after treatment with rt-PA (50nM) + plasminogen (40nM) without or with GM6001 (10μg/ml). GM6001 was inert on its own and did not attenuate rt-PA /plgn-induced shape changes of mouse astrocytes, suggesting that this effect is MMP-independent. n = 2. Scale bars represent 100μm and 40μm in the X10 and X40 images, respectively. (C) GM6001 (10μg/ml), supplemented into the developing buffer of a gelatine zymography, effectively blocks active MMP-2 in conditioned medium of mouse astrocytes as well as MMP-2 and MMP-9 produced by HT1080 human fibrosarcoma cells. (TIF) [file pone.0177332.s004.tif]
